# A combined nasolabial and infra-nasal bi-lobe flap design with double transposition for reconstruction of the ipsilateral upper and lower lips associated with the commissure defect

**DOI:** 10.1051/bmdcn/2019090429

**Published:** 2019-11-14

**Authors:** Nyimi Bushabu Fidele, Yifang Zhao, Wu Tianfu, Yanfang Sun, Bing Liu

**Affiliations:** 1 Department of Oral Maxillofacial Head and Neck Oncology Surgery, School, and Hospital of Stomatology, Wuhan University Wuhan 430079 China; 2 The State Key Laboratory Breeding Base of Basic Science of Stomatology & Key Laboratory of Oral Biomedicine Ministry of Education, School & Hospital of Stomatology, Wuhan University Wuhan 430079 China; 3 Stomatology and Maxillofacial Surgery, Department of Dental medicine, Faculty of Medicine, University of Kinshasa Kinshasa XI 834 DR Congo

**Keywords:** Adjacent bi-lobe flap, Double transposition flap, Asians, Lips defect, Commissure defects, Reconstruction

## Abstract

Several classical flap variations have been designed to reconstruct lip and commissure defects. Although most of these variations maybe the best option for repairing defects, there is an increasing risk of anatomic distortion and functional disability, mostly in older persons. Herein, we present a combined nasolabial and infra-nasal bi-lobe flap design with double transposition, which was used to concomitantly close the upper and lower lips associated with commissure defects.

## 1. Introduction

The lips have important functional and aesthetic roles and dynamically determine the overall impression of the overtone of facial expression. Consequently, disturbance in their dynamics may lead to an exaggerated distortion of the middle and lower face regions. Defects may result from congenital anomalies, trauma, wide local excision for neoplasm, or other inciting events [1]. However, several factors pose a challenge to lip defect reconstruction. Herein, we present a double lobe flap design that combined nasolabial and infra-nasal lobes and double transposition for closing the upper and lower lips associated with commissure defects without secondary aesthetic and functional problems.

### 2. Clinical study

**Fig. 1:**
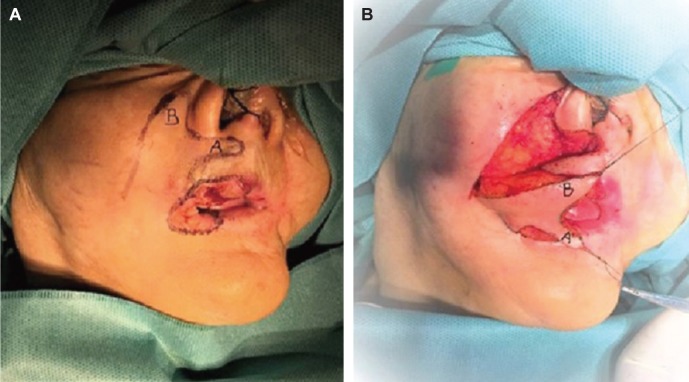
(A). View of the ulcerating painless lesion and two incisions line; one is extended along the anger lines of the cheek and the other is paranasal. (B). According to the pre- analysis for the possibility of the defect, the right commissure and right upper lateral and lower lip defects after lesion resection would belong to the infra-nasal medial plus ipsilateral commissure lip defect. Thus, at the circular outline of the lesion, two incisions were made extending along the Langer’s lines on the cheek and perinasal area, which passed and skirted from the circular base of the ala of the nose and were limited near philtrum. Then, an adjacent double-lobe flap compressing the infra-nasal upper lateral lip lobe (named A) and para-nasal lobe (named B) was raised. After gaining adequate mobilization (Figure 1.B), the combined transposition of lobe A and lobe B closed this defect. The nasolabial lobe closed the upper lateral lip lobe with half of the commissure part of the defect through transposition, and the infra-nasal lobe covered half of the commissure and the lower lip part. For matching the extension with combined transposition and easily accommodating the double transposition of two lobes, A and B, the common pedicle base of these lobes was elongated a little upward to the commissure of the upper and lower lips. In addition, lobes A and B were incised circularly in order to get the correct anatomical shape of the new commissure. The secondary defect or the donor site was closed primarily with advanced surrounding tissues.

**Fig. 2:**
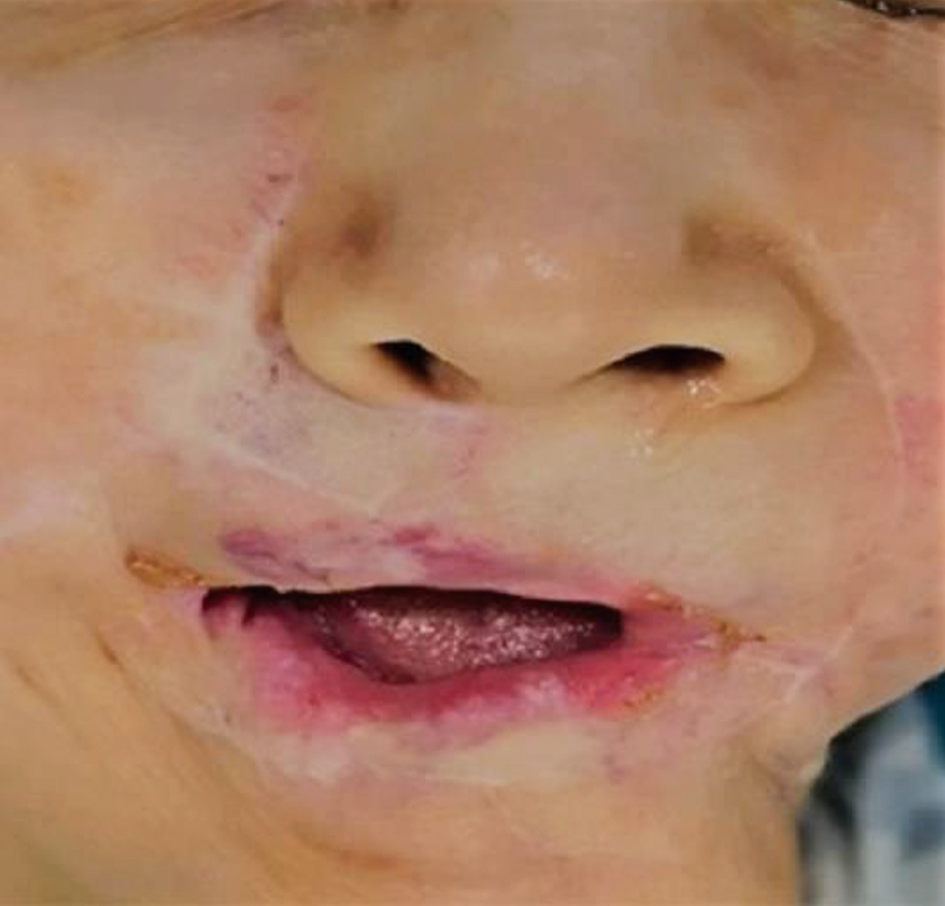
Nine months postoperatively.

An 81-year-old woman that had undergone previous reconstruction for the left lip defect, was admitted in our Department with an ulcerating painful lesion involving the right lip commissure and right half of the upper lip and lower lip, measuring 0.4 cm × 0.6 cm in size (Figure 1A). After excision of the lesion, the defect was repaired with an adjacent double-lobe flap compressing the infra-nasal upper lateral lip lobe (named A) and paranasal lobe (named B) like shown in Figure 1B. The wounds healed uneventfully, and a satisfactory outcome was observed 9 months postoperatively (Figure 2). The pathological diagnosis of the lesion was pseudo-epitheliomas hyperplasia.

## 3. Discussion

Lip reconstruction remains a challenge, and esthetics and donorsite morbidity have become critical considerations in reconstruction; this is particularly relevant in central facial reconstruction. Several aesthetic units are intricately controlled by a complex series of muscles and a dynamic equilibrium exists between the opposing lips; therefore, muscles must be properly restored with an effective reconstruction approach after occurrence of defects.

The lack of any substantial fibrous framework increases the risk of anatomic distortion through wound contraction and, hence, leads to poor functional and esthetic outcomes [2]. The quality of the skin and mucosa of the lips is difficult to match with that of distant flaps; hence, local tissues provide the best results [3]. Various classical flaps have been used worldwide for lip reconstruction, including the Gillies fan flap, Karapandzic flap, Bernard-Burow-Weber flap, Jackson Technique, Abbé-Estlander flap and nasolabial flap, which reflect the overall inadequacy to suit every patient with any given defect [4–7]. To date, fullthickness skin graft, vascularized free flap, and adjacent flap are used for the repair of various lip defects. The adjacent flap that has a variety of designs which respect to the matched color, texture, and thickness of the defect area has been widely recommended. Most of these flaps may be the best choice for repairing lip defects; however, they are somewhat complicated to operate and require more incisions. Moreover, Asian patients have a higher tendency for scar formation after facial surgery; therefore, these techniques should be used secondarily [8]. In addition, skin laxity; sometimes makes repairing of certain defects convenient, and flaps mostly used in elderly patients are not applicable in young patients because of the aesthetic problems [9, 10]. Upper or lower lip defects and commissure defects can be reconstructed using an inferiorly based nasolabial flap, which is an excellent source of local tissue. Therefore, a double flap design combining nasolabial and infra-nasal lobes with double transposition can be used as for reconstruction of ipsilateral upper and lower lip defects in elder women. This double flap is a cutaneous axial flap based on angular artery perforator, if based superiorly or a facial artery perforator, if based inferiorly, as in the present study [11]. Even with other myriad reconstructive options for surgeons, the present double flap design is more reliable for restoring aesthetic and functional aspects of the lip. This design may be the least morbid reconstruction method for upper and lower lips associated with commissure defects compared to other various classical flaps [4–7], and the mean advantage of this flap is the double transposition, double lobes with a common pedicle base in which the upper and lower lips associated with commissure defects can be concomitantly closed. The circular incision between the lobes A and B help to get a good anatomical shape of the new commissure. Thus, the flap design was aesthetically oriented and uncomplicated.

In conclusion, this study presents another alternative option for the reconstruction of lip defects, especially in Asian subjects of advanced age. The flap provides good colour and texturematched tissue to the upper and lower lips. Excellent blood supply based on the facial arteries and the natural appearing scar at the donor site reinforce this flap as a useful adjunct in the reconstruction of the lip and commissure.
